# The Evolution of Implant-Based Breast Reconstruction: Innovations, Trends, and Future Directions

**DOI:** 10.3390/jcm13237407

**Published:** 2024-12-05

**Authors:** Chris Amro, Thomas J. Sorenson, Carter J. Boyd, Kshipra Hemal, Nicholas A. Vernice, Jenn J. Park, Oriana D. Cohen, Mihye Choi, Nolan S. Karp

**Affiliations:** Hansjörg Wyss Department of Plastic Surgery, New York University Langone Health, New York, NY 10016, USA

**Keywords:** breast reconstruction, direct-to-implant, implant, prepectoral placement, synthetic mesh, neurotization, acellular dermal matrix

## Abstract

**Background/Objectives:** Implant-based breast reconstruction has been essential since the 1960s, offering a faster and less invasive alternative to autologous reconstruction. Recent innovations—including direct-to-implant (DTI) reconstruction, advancements in surgical planes, synthetic meshes, and nipple-areolar complex (NAC) neurotization—have improved patient outcomes. This review explores these developments, analyzing their impact on breast reconstruction over the past two decades. **Methods**: A comprehensive literature review was conducted using PubMed, Google Scholar, and Cochrane Library databases, focusing on peer-reviewed studies published up to 2024. Articles were selected based on relevance, quality, and documentation of clinical outcomes and patient satisfaction. **Results**: Findings indicate that DTI reconstruction reduces the need for multiple surgeries, especially in cases with sufficient mastectomy flap quality. Prepectoral placement showed benefits in postoperative comfort and recovery speed compared to subpectoral placement, but had specific risks, such as implant rippling. Synthetic meshes improved implant support and reduced complication rates, while neurotization has shown potential in restoring sensation to the nipple-areolar complex (NAC), addressing quality-of-life concerns. **Conclusions**: Innovations like DTI, prepectoral techniques, and adjunctive mesh and neurotization strategies are advancing patient outcomes. Future research should refine these methods, aiming to expand applicability and further improve aesthetic and sensory outcomes for breast cancer survivors.

## 1. Introduction

Breast cancer is the most common malignancy diagnosed among women in the United States, affecting approximately one in eight women in their lifetime [1]. With advancements in cancer treatments and surgical techniques, breast reconstruction has become an essential part of post-mastectomy care, with implant-based reconstruction being the most common reconstructive option for patients [2]. Over time, implant-based breast reconstruction has evolved significantly, driven by advancements in mastectomy techniques, reconstructive options, and patient expectations.

Historically, the reconstructive surgeon had little involvement in the type of mastectomy performed or location of incisions for the procedure. However, recent years have seen a collaborative approach develop between breast surgeons performing the mastectomy or lumpectomy and plastic surgeons performing the reconstruction. This collaboration aims to optimize both aesthetic and oncological outcomes, a concept referred to as “oncoplastics” [3]. The oncoplastic approach allows for a more personalized treatment plan that balances the patient’s oncological needs with aesthetic considerations.

Since its description in 1972 by John Madden, the current standard for radical mastectomy is the most invasive option [4]. However, the evolution of non-surgical management of breast cancer, including chemotherapies, radiotherapies, and hormone therapies, has enabled increasingly more conservative surgical treatments depending on the size and location of the cancer, which has, in turn, increased the aesthetics of the reconstructed result [5,6,7]. When indicated, the skin-sparing mastectomy (SSM) provides more native breast skin for subsequent reconstruction and the nipple-sparing mastectomy (NSM) provides a similarly large skin envelope while also preserving the nipple-areolar complex (NAC). While some groups have upheld the NSM as an ideal mastectomy with demonstrated high rates of patient satisfaction [8], other reports suggest that SSM followed by NAC reconstruction is a favorable alternative to NSM [9,10].

Increasingly, to achieve optimal aesthetic outcomes and meet the expectations of patients, a collaborative approach between the plastic surgeon and breast surgeon has become essential. The plastic surgeon must work closely with the breast surgeon, not only during the operative procedure but also in preoperative counseling and operative planning. This partnership allows for more precise decision-making and better outcomes, particularly in determining incision locations, mastectomy techniques, and reconstruction plans that balance oncological safety with aesthetic results.

The field of implant-based breast reconstruction has seen significant advancements, particularly with the development of direct-to-implant (DTI) reconstruction, the use of biologic and synthetic meshes, and innovations in neurotization techniques aimed at restoring sensation to the NAC. However, despite these innovations, established techniques such as staged reconstructions and subpectoral implant placements continue to play a critical role in certain clinical scenarios, offering a tailored approach based on individual patient needs. As a result, the evolution of breast reconstruction has broadened the array of available options, allowing for both traditional and modern techniques to be utilized depending on the complexity of each case. This review will explore these developments, focusing on the progression of DTI vs. staged reconstructions, implant placement strategies, mesh usage, and neurotization techniques.

## 2. Materials and Methods

A comprehensive literature review was conducted using multiple databases, including PubMed, Google Scholar, and Cochrane Library Databases to ensure a broad and thorough search. The search focused on articles published up to 2024, to capture the most relevant advancements in implant-based breast reconstruction. Keywords included “DTI breast reconstruction”, “direct-to-implant reconstruction”, “breast implants”, “two-stage breast reconstruction”, “prepectoral breast reconstruction”, “subpectoral breast reconstruction”, “synthetic mesh”, “non-synthetic mesh”, “acellular dermal matrix (ADM)”, and “neurotization”.

Articles were selected based on their relevance to the primary themes in implant-based breast reconstruction, with an emphasis on peer-reviewed journals. Both synthetic meshes and biologic materials, including ADM, were reviewed, as these have played significant roles in shaping modern reconstructive approaches. To provide a comprehensive perspective, landmark studies that marked key advancements in the field—such as the transition from staged to direct-to-implant procedures and the evolution of implant materials—were also included.

All articles were reviewed independently by two authors (TS and CA) to ensure thoroughness and accuracy in selection. This review aims to present a well-rounded analysis of both established and emerging techniques in implant-based breast reconstruction, focusing on the comparative effectiveness of different strategies, the role of soft tissue support materials, and recent innovations, like neurotization, aimed at improving patient outcomes.

## 3. Discussion

### 3.1. Direct to Implant Versus Staged Reconstruction

Historically, breast reconstruction was primarily performed as a two-stage procedure. This approach involved the placement of a tissue expander (TE) at the time of mastectomy, followed by a second-stage surgery to exchange the expander for a permanent implant. Cordeiro et al. demonstrated that staged reconstructions provided reliable long-term results but required multiple procedures [2]. However, as mastectomy techniques evolved, improvements in skin flap quality and a deeper understanding of patient outcomes paved the way for advancements in breast reconstruction techniques. One of the major limitations of the two-stage procedure is the extended treatment timeline, which led to the development of DTI reconstruction as a one-stage alternative.

In DTI reconstruction, the permanent breast implant is placed immediately following mastectomy, eliminating the need for staged surgery. The ideal patient for DTI prepectoral reconstruction generally has well-vascularized thick skin flaps, minimal comorbidities such as diabetes or active smoking, and desires a comparable breast size. This technique has gained traction, particularly in a select group of patients with good skin flap quality and when there is minimal concern for mastectomy flap necrosis. Clinical assessment of the skin flaps is crucial in determining the feasibility of DTI reconstruction. While this has traditionally been conducted through physical examination, technological advances like intraoperative imaging tools, such as the SPY system, are increasingly used to assess flap perfusion and minimize the risk of necrosis, which can lead to complications including implant loss [11,12]. Assessment of the mastectomy flaps with this method requires avoiding epinephrine containing local anesthesia and may not be available in all practice settings given the cost of machinery.

Despite the advancements in DTI reconstruction, staged procedures remain a necessary option for certain patient populations. For patients with compromised skin due to previous radiation therapy, or those who desire a significantly larger reconstruction than the available breast envelope allows, the two-stage approach continues to be the preferred method. Furthermore, for cases where intraoperative assessments raise concerns about skin flap viability, a tissue expander provides a safer, more gradual option for reconstruction. Both approaches—DTI and staged re-constructions—are essential tools, with the decision between the two being highly individualized based on patient-specific factors and intraoperative findings. 

Frey et al. highlighted that DTI has become increasingly popular in nipple-sparing mastectomies, as it offers immediate aesthetic outcomes and reduces the overall number of surgeries, leading to higher patient satisfaction [13,14]. However, it is essential to emphasize that while DTI has provided a less invasive alternative for a select group of patients, the two-stage method remains a critical option in cases where patient safety or aesthetic considerations necessitate a more cautious approach (Table 1).

### 3.2. Evolution of Breast Implants

Breast implants are available in two primary fill materials: saline and silicone. Since the Food and Drug Administration (FDA) moratorium was lifted in 2006 [15], the use of silicone implants has steadily increased, with data indicating higher patient satisfaction compared to saline implants [16]. This preference can be attributed to several factors. First, saline implants, which are filled with sterile saline, tend to have a less natural feel compared to silicone implants. Second, in the event of rupture, saline implants can result in visible asymmetry as the saline is absorbed by the body. Finally, saline implants are more prone to causing noticeable skin rippling, particularly when placed in the prepectoral plane. The evolution of saline implants now includes a subcategory called “structured saline implants” which contain an inner structuring to provide a more natural feel, which have been found in long-term studies to have high patient and surgeon satisfaction, a low rate of capsular contracture, and a low rate of rupture/deflation [17].

The alternative is a silicone breast implant, which has the benefit of feeling more like natural breast tissue due to the increased cohesivity of the silicone gel. These implants must be monitored with imaging to detect rupture, as the silicone elastomer is not absorbed by the body. In the event of a rupture, asymmetries may occur due to the silicone leak into the breast pocket, which may lead to noticeable changes in the shape or feel of the breast. Further evolution of these implants has resulted in “gummy bear silicone implants”, which are form stable with thicker silicone consistency resulting in a firmer breast implant that will maintain shape even if the im-plant shell is fractured.

The surface of breast implants can be either a smooth or textured coating. Initially, im-plants were created with a smooth shell. This provided minimal friction between the implant and the breast pocket, so the implants could shift within that space leading to aesthetic deformities and possible seroma formation. Textured implants were then developed to increase friction between the implant and the breast pocket and prevent micro-shifting of the prosthetic. An added benefit of the texturing on the surface of the implant identified in retrospective studies was a reduced risk of capsular contracture compared to smooth-surfaced implants [18].

However, the texturing on these implants was soon found to be associated with the development of breast implant-associated anaplastic large cell lymphoma (BIA-ALCL), resulting in an FDA recall on most textured breast implants. As such, most textured devices are no longer available for use in the United States, and most breast implants that are placed in the United States are manufactured with a smooth shell [19]. Despite this, implants with micro-texturing—a less aggressive form of surface texturing—continue to be manufactured and remain FDA-approved. These micro-textured implants are designed to offer some of the benefits of texturing—such as increased friction to reduce implant mobility—while minimizing the risks associated with more aggressive surface textures. Recent studies have reported outcomes in terms of both aesthetic results and highlighted the promising safety profile of micro-textured implants [20]. A study by Sforza et al. demonstrated that micro-textured implants achieved satisfactory aesthetic outcomes with reduced capsular contracture rates and no association with BIA-ALCL [21]. Similarly, De Boer et al. highlighted the advantages of micro-textured implants, stating that they provide an effective compromise between smooth and textured implants, delivering improved outcomes without the heightened risks [22].

Although not FDA-approved in the United States, Polyurethane-coated implants have been used globally and are noted for their ability to reduce capsular contracture further while offering excellent implant stability [18,23]. These implants provide a unique option for patients in countries where they are available.

### 3.3. Evolution of Implant Placement

The plane of breast implant placement is a principal consideration for breast reconstruction and must be considered in conjunction with the mastectomy performed by the breast sur-geon. The options include the prepectoral plane, the submuscular plane, or the dual-plane in which the breast implant is partially submuscular and partially in the prepectoral plane. Each plane has its advantages and disadvantages, but the location has been found to be largely equivalent in terms of reconstructive value [24,25]. The choice of plane is a collaborative decision between the patient and surgeon, based on individual circumstances and preferences. However, the final decision is often made intraoperatively, depending on the vascularity of the mastectomy flaps.

The submuscular (or subpectoral) plane was traditionally the most frequently utilized approach for breast reconstruction. By positioning the implant beneath the pectoralis major muscle, this technique offers several advantages, including providing vascularized tissue in cases of compromised mastectomy flaps, creating a natural contour including a sloped upper pole, and minimizing complications like capsular contracture. However, this approach is not without its limitations. Nahabedian et al. found that subpectoral placement reduces the risk of implant visibility and palpability, but often at the cost of muscle-related complications like animation deformity [26].

In contrast, the prepectoral approach, which places the implant above the pectoralis muscle, has emerged as an alternative that avoids many of the muscle-related complications of submuscular placement. Reitsamer et al. were among the early advocates for prepectoral placement, reducing postoperative pain and animation deformity [27]. This technique was particularly effective when combined with acellular dermal matrices (ADMs), which provide additional support to the overlying skin. Though the prepectoral plane has the advantage of avoiding morbidity associated with pectoralis muscle elevation, it may carry at heightened risk of major complications secondary to flap necrosis or incisional dehiscence. Nelson et al. found that prepectoral techniques resulted in reduced postoperative pain and quicker recovery, though there was a slight increase in seroma formation [28]. For patients with a history of neoadjuvant radiation/chemotherapy or requiring adjuvant radiation/chemotherapy in the future, either the prepectoral or submuscular plane have been found to be feasible reconstructive options [29,30,31].

Ultimately, the decision between prepectoral and submuscular placement depends on various patient-specific factors, including skin flap quality, absence of prior radiation, risk of necrosis, patient’s tolerance for potential complications, and surgeon preference. While submuscular placement remains a reliable choice, especially in patients at higher risk of skin flap compromise, prepectoral techniques, particularly when combined with acellular dermal matrices (ADMs), are increasingly being utilized due to their advantages in reducing pain and recovery time while maintaining comparable aesthetic outcomes. The importance of careful patient selection and individualized treatment planning cannot be overstated when determining the most appropriate reconstruction technique.

Dual-plane implant placement is a hybrid of the two previously discussed techniques that involves elevation of the pectoralis muscle and superior muscular coverage of the breast *prosthetic* without elevating or securing the serratus fascia over the implant. As this leaves the *inferolateral* aspect of the breast implant directly adjacent to the mastectomy flap, soft tissue support is frequently used to provide an inferolateral sling that controls the position of the implant on the chest wall and prevents displacement of the implant with pectoralis muscle contraction [32].

In addition to these approaches, lipofilling has emerged as an effective adjunct technique in implant-based reconstruction. By using autologous fat grafting, lipofilling addresses common issues such as implant rippling, visibility, and irregular contours [33,34].

### 3.4. Use of Mesh in Reconstruction

One of the recent important technological advances in implant-based breast reconstruction is the development of materials to use for soft tissue support [35]. These include biologic mesh, synthetic mesh, and acellular dermal matrix (ADM) [36]. There is a paucity of data supporting the use of one soft tissue support option over another [37,38,39,40,41]. However, it is important to note that all of these products are used off-label, in the support of a breast implant as there is limited FDA market approval for these materials [41].

The introduction of synthetic meshes in breast reconstruction has provided surgeons with more versatile tools to support implants, particularly in prepectoral breast reconstruction. Breuing et al. were pioneers in using mesh slings to support implants in a single-stage reconstruction procedure, reducing the need for multiple surgeries [42]. Their technique has since evolved, with synthetic meshes providing the same benefits while minimizing the risk of complications. Gschwantler-Kaulich et al. conducted a comparative study between synthetic meshes and ADMs, showing that synthetic meshes, particularly in the subpectoral plane, resulted in fewer complications rates compared to ADMs, with implant extrusion rates at 7.7% vs. 30.4% and overall complication rates at 24% vs. 39.1% [37]. Similarly, Tessler et al. noted that synthetic meshes provide similar aesthetic outcomes to ADMs while being more cost-effective, reducing direct material costs up to $172,112 over 10 months and low complication rates of 6.6% [43]. Furthermore, Clark et al. conducted the most recent systematic review and meta-analysis in the literature, analyzing data from eight comparative studies. Their findings revealed no difference in the risk of infection between synthetic meshes and ADMs but demonstrated a reduced risk of re-operation or explant with synthetic meshes [44]. Still, ADMs remain a popular option given their demonstrated efficacy in improving breast implant position though their use is associated with higher costs and risk of seroma and infection (Table 2) [45,46,47,48,49,50,51].

The practice of the senior authors has evolved over the years, and they currently utilize poly-4-hydroxybutyrate mesh to circumferentially wrap the implant and create a pseudo “textured” breast implant with a cuff to be used for securing the breast implant in the breast pocket [52]. This approach has been highly effective in providing additional stability to the implant, further optimizing direct-to-implant breast reconstructions. Despite the constant evolution of techniques with soft tissue support, it is important to note that no single product or technique has definitive superiority. Good results have been reported across a variety of soft tissue support materials and approaches, underscoring the importance of individualized patient care and the surgeon’s expertise in selecting the most appropriate method for each case.

### 3.5. Neurotization: Addressing Sensory Loss

One of the key challenges in breast reconstruction has been the loss of sensation in the NAC. Likely the hottest topic in breast reconstruction at the present moment is the push to neurotize the NAC following NSM. Reports have documented that the decrease in sensation of the mastectomy flap and NAC following an NSM had been identified as a source of substantial patient dissatisfaction [53]. This process involves the identification of the third, fourth, or fifth lateral intercostal nerves as they emerge from the chest wall and coaptation with a nerve allograft to the NAC. Peled et al. were among the first to demonstrate that neurotization—reconnecting the intercostal nerves to the NAC—could restore sensation in patients undergoing immediate implant reconstruction [54]. This technique involves using nerve allografts to bridge the gap from the cut lateral intercostal nerve to the NAC during reconstruction, offering the potential for sensory recovery in previously numb areas. Tevlin et al. further supported the use of neurotization, showing patients experienced some degree of sensory recovery following this technique [55]. The ability to preserve or restore sensation offers a significant improvement in quality-of-life for breast cancer survivors. Early data are promising, though long-term outcomes have not yet confirmed the efficacy of these measures [54,55]. Results should be interpreted with caution as techniques and outcomes are not yet standardized.

### 3.6. Future Direction

The field of implant-based breast reconstruction continues to evolve, driven by advances in biomaterials, surgical techniques, and personalized patient care. Future research should focus on the development of next-generation biomaterials, including synthetic and biologic meshes with improved integration, lower complication rates, and cost-effectiveness, to optimize outcomes for diverse patient populations. Refined techniques of neurotization, aimed at restoring sensation to the nipple-areolar complex, require further long-term studies to establish their efficacy and standardize approaches for broader application. Additionally, fine-tuning patient selection guidelines through predictive tools and data-driven decision-making will enhance the ability to match reconstructive techniques—such as direct-to-implant vs. staged reconstruction or prepectoral vs. subpectoral placement—with individual patient needs, particularly in complex cases involving radiation or compromised tissue. By addressing these key areas, the field can advance toward achieving superior aesthetic, functional, and quality-of-life outcomes, ensuring a more tailored and effective approach to breast reconstruction.

## 4. Conclusions

The evolution of implant-based breast reconstruction reflects ongoing advancements in surgical techniques and materials. DTI reconstruction has reduced the need for multi-stage procedures, though there are still limitations in its applicability, particularly for patients with compromised skin flaps. Similarly, while prepectoral implant placement has minimized complications associated with muscle dissection, it presents its own set of challenges, such as increased risks of implant rippling and skin flap necrosis. Synthetic meshes have provided a cost-effective and reliable alternative to biologic meshes, improving outcomes in both prepectoral and subpectoral reconstructions. Finally, neurotization techniques offer the potential to restore sensation, addressing a critical quality-of-life issue for many patients. Future research will likely focus on refining these techniques, reducing complications, and providing more reliable aesthetic results. This ongoing innovation ensures that implant-based breast reconstruction will continue to evolve and improve out-comes for breast cancer survivors.

## Figures and Tables

**Table 1:** Key assessments and considerations for DTI, two-stage, prepectoral, and subpectoral approaches to guide both preoperative and intraoperative decision-making. Importantly, reconstruction type (one-stage vs. two-stage) and plane placement (prepectoral vs. submuscular) require an individualized approach considering each patient’s reconstructive goals and the unique patient history and clinical scenario. The above guidelines serve as a flexible guide rather than rigid rules for reconstructive planning.

Phase	Parameters	DTI Considerations	Two-Stage Considerations	Prepectoral Considerations	Subpectoral Considerations
**Preoperative**	Patient goals	Desire to be a similar size to preop	Desire to be larger in size	Low tolerance for animation deformity	Low tolerance for rippling
	Comorbidities	Minimal comorbidities (e.g., no smoking, non-diabetic)	Higher tolerance for patients with more comorbidities (e.g., smoking, diabetic)	Minimal comorbidities (e.g., no smoking, non-diabetic)	Higher tolerance for patients with more comorbidities (e.g., smoking, diabetic)
	History of radiation	Safer without a history of prior radiation	Preferred approach with history of prior radiation	Safer without a history of prior radiation	Preferred approach with history of prior radiation
	Anticipated post-mastectomy radiation therapy	Equivocal	Equivocal	Safer with robust mastectomy flap	Higher rate of capsular contracture
**Intraoperative**	Mastectomy Flap quality				
	Good flap quality	Ideal scenario	Acceptable option	Ideal scenario	Remains an option patient on patient goals/remainder of history
	Compromised flap quality	Not suitable in prepectoral plane; could consider subpectoral	Preferred	Not suitable	Option pending location of flap compromise; may require serratus fascia for inferolateral coverage

**Table 2:** Differences between synthetic and biologic meshes used for soft tissue support in breast reconstruction. ADM: Acellular dermal matrix. *: statistically significant. ^†^: not statistically significant.

Factor	Synthetic Mesh	Biologic Mesh (ADM)
Implant extrusion rate	Lower than ADM *	Higher than synthetic mesh *
Overall complication rate	Lower than ADM ^†^	Higher than synthetic mesh ^†^
Cost	Cost-effective	Significantly higher
Aesthetic outcomes	Comparable to ADM	Comparable to synthetic mesh
